# *Radiation Oncology* reviewer acknowledgement 2014

**DOI:** 10.1186/s13014-015-0328-5

**Published:** 2015-02-22

**Authors:** Claus Belka, Thalyana Smith-Vikos

**Affiliations:** Department of Radiation Oncology, University Hospital of Munich, Marchioninistr. 15, 81377, Munich, Germany; BioMed Central, Floor 6, 236 Gray’s Inn Road, London, WC1X 8HB UK

## Abstract

The editorial team of *Radiation Oncology* would like to thank all our reviewers who have contributed to the journal in Volume 9 (2014).

**Yasser Abo-Madyan**

Germany

**Sebastian Adamczyk**

Poland

**Dario Agnese**

Italy

**Merina Ahmed**

United Kingdom

**Mario Airoldi**

Italy

**Alireza Akhondi-Asl**

United States of America

**Markus Alber**

Denmark

**Francesca Albertini**

Switzerland

**Valerie Albrecht**

Germany

**Manuel Algara**

Spain

**Filippo Alongi**

Italy

**Mohamad Al-Sheikhly**

United States of America

**Natasa Anastasov**

Germany

**Nicolaus Andratschke**

Switzerland

**C Nicolaj Andreassen**

Denmark

**Mekhail Anwar**

United States of America

**Meritxell Arenas**

Spain

**Dirk Arnold**

Germany

**Vasileios Askoxylakis**

Germany

**Myriam Ayadi**

France

**Omid Azimzadeh**

Germany

**Woo Kyun Bae**

South Korea

**Amit Bahl**

India

**Kurt Baier**

Germany

**Rinske Bakker**

Netherlands

**Hendrik Ballhausen**

Germany

**Dimos Baltas**

Germany

**James Balter**

United States of America

**Robyn Banerjee**

Canada

**Mark Bangert**

Germany

**Philip Bardin**

Australia

**Zarko Barjaktarovic**

Germany

**Chris Barker**

United States of America

**Guido Baroni**

Italy

**Tristan Barrett**

United Kingdom

**Michael Barton**

Australia

**Brigitta Baumert**

Germany

**Beth Beadle**

United States of America

**Sergio Benavente**

Spain

**Bernhard Berger**

Germany

**Eric Berthelet**

Canada

**Dirk Beutner**

Germany

**Albert Biete**

Spain

**Mario Bignardi**

Italy

**Tina Binderup**

Denmark

**Wolfgang Birkfellner**

Austria

**Marc Bischof**

Germany

**Pierre Blanchard**

France

**Oliver Blanck**

Germany

**Sanne Blinde**

Netherlands

**Andreas Block**

Germany

**Felix Böckelmann**

Germany

**Judit Boda-Heggemann**

Germany

**Stefan Boeck**

Germany

**Edwin Boelke**

Germany

**Beat Bojaxhiu**

Switzerland

**Fabrice Bonnet**

France

**Alp Özgün Börcek**

Turkey

**Tom Boterberg**

Belgium

**Dirk Bottke**

Germany

**Ugo Bottoni**

Italy

**Michael Brada**

United Kingdom

**Julie Bradley**

United States of America

**Herbert Braselmann**

Germany

**Michael Bremer**

Germany

**Giuseppe Brisinda**

Italy

**James Brown**

United Kingdom

**Paul Brown**

United States of America

**Gregor Bruggmoser**

Germany

**Thomas Brunner**

Germany

**Vicente Bruzzaniti**

Italy

**Wilfried Budach**

Germany

**Krzysztof Bujko**

Poland

**Ralph Bundschuh**

Germany

**Jan Bussink**

Netherlands

**Karl Butterworth**

United Kingdom

**Felipe Calvo**

Spain

**Mauricio Campos Daziano**

Chile

**Aras Emre Canda**

Turkey

**Lynnette Cary**

United States of America

**Richard Castillo**

United States of America

**Annemieke Cats**

Netherlands

**Charles Catton**

Canada

**Jimmy Caudell**

United States of America

**Jean-Michel Caudrelier**

Canada

**Guillaume Cazoulat**

France

**Laura Cella**

Italy

**Francesco Cellini**

Italy

**Laura Cerezo**

Spain

**Anuradha Chakravarthy**

United States of America

**Marc Chamberlain**

United States of America

**Annie Chan**

United States of America

**Albert Chang**

United States of America

**Joe Chang**

United States of America

**Ming-Chih Chang**

Taiwan

**Samuel Chao**

United States of America

**Naved Chaudhri**

Germany

**Buxin Chen**

United States of America

**Delphine Chen**

United States of America

**Ming Chen**

China

**Ronald Chen**

United States of America

**Ui-Kyu Chi**

South Korea

**Kwan-Hwa Chi**

Taiwan

**Kevin Choe**

United States of America

**Hans Christiansen**

Germany

**Daniel Chua**

Hong Kong

**Michael Chuong**

United States of America

**Nikola Cihoric**

Switzerland

**Savino Cilla**

Italy

**Mario Ciocca**

Italy

**Alessandro Clivio**

Switzerland

**Anthony Cmelak**

United States of America

**Sean Collins**

United States of America

**Rob Coppes**

Netherlands

**Gennaro Cormio**

Italy

**Luca Cozzi**

Italy

**Gilles Crehange**

France

**Hans Crezee**

Netherlands

**M.A. Cuesta**

Netherlands

**Johan Peter Cuijpers**

Netherlands

**Fred Currell**

United Kingdom

**Alan Dal Pra**

Switzerland

**Megan Daly**

United States of America

**Sidsel Damkjaer**

Denmark

**Rolando M. D'Angelillo**

Italy

**Indra Das**

United States of America

**Prajnan Das**

United States of America

**Gert De Meerleer**

Belgium

**Antonella De Rosa**

Italy

**Armando De Virgilio**

Italy

**letizia deantonio**

Italy

**Joseph Deasy**

United States of America

**Melanie Deberne**

France

**Robert Den**

United States of America

**Carmen Dence**

United States of America

**Timm Denecke**

Germany

**Francesco Deodato**

Italy

**Beatrice Detti**

Italy

**Andreas Dinkel**

Germany

**Oliver Dohm**

Germany

**Jacobson Don**

United States of America

**Qinghua Dong**

China

**Dimitrios Dougenis**

Greece

**Stephen Dowdell**

Australia

**Weiliang Du**

United States of America

**Yadranko Ducic**

United States of America

**Marciana Nona Duma**

Germany

**Peter Dunscombe**

Canada

**Fréderic Duprez**

Belgium

**Marco Durante**

Germany

**Raghav Dwivedi**

United Kingdom

**Yvonne Dzierma**

Germany

**Thomas Eade**

Australia

**Michael Eble**

Germany

**Franziska Eckert**

Germany

**Monika Ecsedy**

Hungary

**Jens Edmund**

Denmark

**Avraham Eisbruch**

United States of America

**Hoda Elbakry**

Egypt

**John Eley**

United States of America

**Olgun Elicin**

Switzerland

**Lorraine Elit**

Canada

**Michael Elliott**

United States of America

**Nagy Elsayyad**

United States of America

**Medhat ElSebaie**

Egypt

**SR Emani**

India

**Svend Engelholm**

Denmark

**Benedikt Engels**

Belgium

**Mustafa Esassolak**

Turkey

**Marion Essers**

Netherlands

**Veni Ezhil**

United Kingdom

**Khashayar Fakhrian**

Germany

**Aurora Fassi**

Italy

**Dimitrios Fatouros**

Greece

**Barry Feig**

United States of America

**Steven Feigenberg**

United States of America

**Pascal Fenoglietto**

France

**Claudio Festuccia**

Italy

**Christian Fiandra**

Italy

**Kathryn Field**

Australia

**Rainer Fietkau**

Germany

**Andrea Riccardo Filippi**

Italy

**Jacob Finkelstein**

United States of America

**Michele Fiore**

Italy

**Alba Fiorentino**

Italy

**Michael K Fix**

Switzerland

**Jochen Fleckenstein**

Germany

**Gerald Fogarty**

Australia

**Antonella Fogliata**

Switzerland

**Emmanouil Fokas**

United Kingdom

**Nicolas Foray**

France

**Kenneth Forster**

United States of America

**Piero Fossati**

Italy

**Eugene Fourkal**

United States of America

**Claudia Fournier**

Germany

**Giovanni Franchin**

Italy

**Nicolaas Franken**

Netherlands

**Thomas Friedrich**

Germany

**Junichi Fukada**

Japan

**Simone Fulda**

Germany

**Christoph Fürweger**

Germany

**Nobukazu Fuwa**

Japan

**Udo Gaipl**

Germany

**Razvan Galalae**

Germany

**Maria Antonietta Gambacorta**

Italy

**Robin Garcia**

France

**Elisabetta Gargioni**

Germany

**Cristina Garibaldi**

Italy

**Laurie Gaspar**

United States of America

**Harriet Gee**

Australia

**Ian Geh**

United Kingdom

**Dietmar Georg**

Austria

**Tawfik Giaddui**

United States of America

**Chiara Gianoli**

Italy

**Iris Gibbs**

United States of America

**David Gierga**

United States of America

**Suki Gill**

Australia

**Frank Giordano**

Germany

**Meredith Giuliani**

Canada

**Christoph Glanzmann**

Switzerland

**Bengt Glimelius**

Sweden

**Rob Glynne-Jones**

United Kingdom

**Ziya Gokaslan**

United States of America

**Daniel Gomez**

United States of America

**Sara González**

Argentina

**Holger Gottschlag**

Germany

**Julie Goudreault**

Canada

**Gerhard Grabenbauer**

Germany

**Christian Graeff**

Germany

**Joel Greenberger**

United States of America

**Christopher Gregory**

United Kingdom

**Daila Gridley**

United States of America

**Perry Grigsby**

United States of America

**Anca Grosu**

Germany

**Peter Gruebling**

Germany

**Tejpal Gupta**

India

**Sarah Gwynne**

United Kingdom

**Jonathan Haas**

United States of America

**Gregor Habl**

Germany

**Scott Hadley**

United States of America

**Matthias Häfner**

Germany

**Christopher Hallemeier**

United States of America

**Gerry Hanna**

United Kingdom

**Gerard G Hanna**

United Kingdom

**Wendy Hara**

United States of America

**Nicholas Hardcastle**

Australia

**Josefin Hartmann**

Germany

**Christina Haston**

Canada

**Birgitte Havelund**

Denmark

**Maria Hawkins**

United Kingdom

**Xia He**

China

**Felix Heinemann**

Germany

**Christian Heinz**

Germany

**Frank Hensley**

Germany

**Klaus Herfarth**

Germany

**Dwight Heron**

United States of America

**Julia Heß**

Germany

**Ekkehard Hewer**

Switzerland

**Reinhard Heyd**

Germany

**Tarek Hijal**

Canada

**Robin Hill**

Australia

**Daisaku Hirano**

Japan

**Ying Hitchcock**

United States of America

**Ivy Ho**

Singapore

**James Hodge**

United States of America

**Frank Hoebers**

Netherlands

**Fatemeh Homaeishandiz**

Iran

**Ulrich Theodor Hopt**

Germany

**Orla Howe**

Ireland

**Morten Høyer**

Denmark

**Feng-Ming Hsu**

Taiwan

**Chaosu Hu**

China

**Weigang Hu**

China

**Maarten CCM Hulshof**

Netherlands

**Dezheng Huo**

United States of America

**Sefik Igdem**

Turkey

**George Iliakis**

Germany

**Hiroshi Inoue**

Japan

**Edy Ippolito**

Italy

**Hassan Iqbal**

Pakistan

**Hiromichi Ishiyama**

Japan

**Fumiaki Isohashi**

Japan

**Jun Itami**

Japan

**Seong Soon Jang**

South Korea

**Marek Janiak**

Poland

**Nathalie Jansen**

Germany

**Maha Jawad**

United States of America

**Supriya Jayant Sastri**

India

**Urszula Jelen**

Germany

**Verena Jendrossek**

Germany

**Alexandra D. Jensen**

Germany

**J. Martin Jensen**

Canada

**Jae-Uk Jeong**

South Korea

**Branislav Jeremic**

Serbia

**Xun Jia**

United States of America

**Esther Jimenez-Jimenez**

Spain

**Hosang Jin**

United States of America

**Meredith Johnston**

Australia

**Peter Johnstone**

United States of America

**Nuria Jornet**

Spain

**Mirjana Josipovic**

Denmark

**German Juan**

Spain

**Alain Jung**

France

**Markus Juonala**

Finland

**Johannes Kaanders**

Netherlands

**Josephine Kang**

United States of America

**Tom Karagiannis**

Australia

**Giorgos Karakousis**

United States of America

**Sana Karam**

United States of America

**Christian P. Karger**

Germany

**Sonja Katayama**

Germany

**Hiroyuki Katoh**

Japan

**David Kaul**

Germany

**Chul-Seung Kay**

South Korea

**Ludwig Keilholz**

Germany

**Monika Keller**

Germany

**Markus Kellermeier**

Germany

**Lucyna Kepka**

Poland

**Ki Chang Keum**

South Korea

**YongKan Ki**

South Korea

**Merrill Kies**

United States of America

**David Kim**

Canada

**Grace Kim**

United States of America

**Jihun Kim**

United States of America

**Jin Sung Kim**

South Korea

**Jaehyup Kim**

United States of America

**Kwang Seok Kim**

South Korea

**Mi-Sook Kim**

South Korea

**Dae Yong Kim**

South Korea

**Su Ssan Kim**

South Korea

**Yongbok Kim**

United States of America

**Young Seok Kim**

South Korea

**Christian Kirisits**

United Kingdom

**Stephan Kloeck**

Switzerland

**Sebastian Klüter**

Germany

**Andrew Kneebone**

Australia

**Lukas Knybel**

Czech Republic

**Thomas Koch**

Germany

**Stefan Koerber**

Germany

**Kiyotaka Kohshi**

Japan

**Masahiko Koizumi**

Japan

**Marisa Kollmeier**

United States of America

**Bridget Koontz**

United States of America

**Gyoergy Kovacs**

Germany

**Josef Kovarik**

United Kingdom

**Takuyo Kozuka**

Japan

**Michael Kraemer**

Germany

**Mechthild Krause**

Germany

**Andra Krauze**

United States of America

**Robert Krempien**

Germany

**Marco Krengli**

Italy

**David Krug**

Germany

**Dmitri Krysko**

Belgium

**Andrzej Kukielka**

Poland

**Shaleen Kumar**

India

**Sanath Kumar**

United States of America

**Pavel Kundrat**

Germany

**Charles Kunos**

United States of America

**Guntram Kunz**

Germany

**Patrick Kupelian**

United States of America

**Abraham Kuten**

Israel

**Young Kwok**

United States of America

**Frank Lagerwaard**

Netherlands

**Tamara Lah**

Slovenia

**Vincent Lai**

Hong Kong

**Rachelle Lanciano**

United States of America

**Stephanie Lang**

Switzerland

**Normand Laperriere**

Canada

**Pedro C. Lara**

Spain

**George Laramore**

United States of America

**Ingmar Lax**

Sweden

**Cathy Lazarus**

United States of America

**Anne Lee**

Hong Kong

**John Lee**

Belgium

**Yun-Sil Lee**

South Korea

**Sebastian Lettmaier**

Germany

**Rolf Lewensohn**

Sweden

**Dadong Li**

United States of America

**Jianbin Li**

China

**Taoran Li**

United States of America

**Zuofeng Li**

United States of America

**Xiaoying Liang**

United States of America

**Charles Limoli**

United States of America

**Jin-Ching Lin**

Taiwan

**Katja Lindel**

Germany

**Bodo Lippitz**

Sweden

**An Liu**

United States of America

**Chun-feng Liu**

China

**Meng-Zhong Liu**

China

**Chen Lixin**

China

**Simon Lo**

United States of America

**Luiz Lobato**

United States of America

**Kristina Loessl**

Switzerland

**Frank Lohr**

Germany

**Jiade Lu**

Singapore

**Shun Lu**

China

**John Lukens**

United States of America

**Martin Lundsgaard Hansen**

Denmark

**LD Lunsford**

United States of America

**L. Dade Lunsford MD**

United States of America

**Yulia Lyatskaya**

United States of America

**Victor Macias**

Spain

**Katsuya Maebayashi**

Japan

**Yoshihiko Maehara**

Japan

**Kevin Maggio**

United States of America

**Dennis Mah**

United States of America

**Karsten Mahnke**

Germany

**Cornelius Maihöfer**

Germany

**Philippe Maingon**

France

**Bettina Maiwald**

Germany

**Raymond Mak**

United States of America

**Chiyoko Makita**

Japan

**Eirik Malinen**

Norway

**Pietro Mancosu**

Italy

**Robert Mandic**

Germany

**Robert Maráz**

Hungary

**Carmelo Marino**

Italy

**Simone Marnitz**

Germany

**Michal Masarík**

Czech Republic

**Laura Masucci**

Canada

**Koji Matsuo**

United States of America

**Yukinori Matsuo**

Japan

**Christiane Matuschek**

Germany

**Riccardo Maurizi Enrici**

Italy

**Andrew McDonald**

United States of America

**Stephen McMahon**

United Kingdom

**James Melotek**

United States of America

**Veerle Melotte**

Netherlands

**Cynthia Menard**

Canada

**Alejandra Mendez Romero**

Netherlands

**Oliver Micke**

Germany

**Gerald Mickisch**

Germany

**Ben Mijnheer**

Netherlands

**Michael Milano**

United States of America

**Giuseppe Minniti**

Italy

**Raymond Miralbell**

Switzerland

**Norio Mitsuhashi**

Japan

**Masahiko Miura**

Japan

**Giulio Modorati**

Italy

**Raphaël Moeckli**

Switzerland

**Simone Moertl**

Germany

**Majid Mohiuddin**

United States of America

**Alessio G. Morganti**

Italy

**Masahiro Morimoto**

Japan

**W. James Morris**

Canada

**Gerard Morton**

Canada

**Lutz Moser**

Germany

**Andre Mouton**

United Kingdom

**Ralph Muecke**

Germany

**Klaus Mueller**

Germany

**Arndt-Christian Müller**

Germany

**Per Munck af Rosenschöld**

Denmark

**Yuji Murakami**

Japan

**Martin Murphy**

United States of America

**Lars Murrer**

Netherlands

**Olaf Nairz**

Austria

**Mitsuhiro Nakamura**

Japan

**Takashi Nakano**

Japan

**Sapna Nangia**

India

**Patrick Naumann**

Germany

**Pierina Navarria**

Italy

**Rodolfo Negri**

Italy

**Ursula Nestle**

Germany

**Nicole Nesvacil**

Austria

**Andrea Ng**

United States of America

**R. Charles Nichols**

United States of America

**Carsten Nieder**

Norway

**Gabriele Niedermann**

Germany

**Peter Niehoff**

Germany

**Yuzuru Niibe**

Japan

**Holly Ning**

United States of America

**Paige Nitsch**

United States of America

**Jenny Nobes**

United Kingdom

**Gianmauro Numico**

Italy

**Joost Nuyttens**

Netherlands

**Jan Nyman**

Sweden

**Uwe Oeh**

Germany

**Toshiyuki Ogata**

Japan

**Shinichi Ohta**

Japan

**Eric Ojerholm**

United States of America

**Didem Colpan Oksuz**

United Kingdom

**Murat Okutan**

Turkey

**Kenneth Olivier**

United States of America

**Ivo Olivotto**

Canada

**Cem Onal**

Turkey

**Michael Orth**

Germany

**Feras Oskan**

Germany

**Piet Ost**

Belgium

**Christian Ostheimer**

Germany

**Mattia Falchetto Osti**

Italy

**Oliver Ott**

Germany

**Natsuo Oya**

Japan

**Mahmut Ozsahin**

Switzerland

**Chiara Paganelli**

Italy

**David Palma**

Canada

**Joshua Palmer**

United States of America

**Jianji Pan**

China

**Valerie Panet-Raymond**

Canada

**Alexandros Papachristofilou**

Switzerland

**Evangelos Pappas**

Greece

**Dillip Kumar Parida**

India

**François Paris**

France

**Paolo Passoni**

Italy

**Abhijit Patel**

United States of America

**Arnold Paulino**

United States of America

**Jacob Pe'er**

Israel

**Cheng Peng**

United States of America

**Dolly Penn**

United States of America

**Vincent Pennaneach**

France

**J.J. Penninkhof**

Netherlands

**Joseph Pepek**

United States of America

**Rodrigo Perez**

Brazil

**Francesco Perri**

Italy

**Nadeem Pervez**

Canada

**Gianfranco Pesce**

Switzerland

**Maria Grazia Petrongari**

Italy

**Heike Peulen**

Netherlands

**Asja Pfaffenberger**

Germany

**Thomas Pfluger**

Germany

**Daniel Pham**

Australia

**Justin Phillips**

United States of America

**Alessia Pica**

Switzerland

**Steffi Ulrike Pigorsch**

Germany

**Karsten Pilones**

United States of America

**Nuno Pimentel**

Portugal

**Michael Pinkawa**

Germany

**Tomasz Piotrowski**

Poland

**Ludwig Plasswilm**

Switzerland

**Julio Plata Bello**

Spain

**George Plataniotis**

United Kingdom

**Volker Platz**

Germany

**Alan Pockley**

United Kingdom

**Wojciech Polkowski**

Poland

**Bruce Pollock**

United States of America

**Bjoern Poppe**

Germany

**Vincent Potiron**

France

**Per Rugaard Poulsen**

Denmark

**Bas Pouw**

Netherlands

**Olivier Pradier**

France

**Heru Prasetio**

Germany

**Brendan Prendergast**

United States of America

**Kevin Prise**

United Kingdom

**Martin Pruschy**

Switzerland

**Newell Pugh**

United States of America

**Paul Martin Putora**

Switzerland

**Florian Putz**

Germany

**Hongryull Pyo**

South Korea

**Ibrahim Qaddoumi**

United States of America

**Hai Qu**

United States of America

**Harvey Quon**

Canada

**Dirk Rades**

Germany

**Ganesh Radhakrishna**

United Kingdom

**Zvi Ram**

Israel

**Giuliano Ramadori**

Germany

**Sara Ramella**

Italy

**Ulla Ramm**

Germany

**Ganesh Rao**

United States of America

**Coen Rasch**

Netherlands

**Andrew Rauth**

Canada

**Michael Reardon**

United States of America

**Anne Hansen Ree**

Norway

**Valerie Reed**

United States of America

**Marc Remke**

Canada

**James Renaud**

Canada

**Swaroop Revannasiddaiah**

India

**Nadeem Riaz**

United States of America

**Marco Riboldi**

Italy

**Umberto Ricardi**

Italy

**Andreas Richter**

Germany

**Christian Richter**

Germany

**Daniel Richter**

Germany

**Jens Ricke**

Germany

**Ron Riesenburger**

United States of America

**Oliver Riesterer**

Switzerland

**Andreas Rimner**

United States of America

**Mark Rivard**

United States of America

**Mack Roach**

United States of America

**David Roberge**

Canada

**Stephen Roberts**

United Kingdom

**Franz Rödel**

Germany

**George Rodrigues**

Canada

**Erik Roelofs**

Netherlands

**Marianne Roenjom**

Denmark

**Dirk Roggenbuck**

Germany

**Teboh Roland**

United States of America

**Olivier Rouvière**

France

**Peter Rubin**

United States of America

**Carmen Rubio**

Spain

**Tatjana Rundek**

United States of America

**Elvio Grazioso Russi**

Italy

**Jean-claude Rwigema**

United States of America

**Mohammadsaeed Saboori**

Germany

**Sotaro Sadahiro**

Japan

**Azmat Sadozye**

United Kingdom

**Arjun Sahgal**

Canada

**Masatoshi Saito**

Japan

**Ladan Saleh-Ebrahimi**

Germany

**Riccardo Santoni**

Italy

**Joseph Santoro**

United States of America

**Naoko Sanuki-Fujimoto**

Japan

**Yoko Satoh**

Japan

**William Sause**

United States of America

**Bryan Schaly**

Canada

**Daniel Schanne**

United States of America

**Daniel Schanne**

United States of America

**Stephan Scheidegger**

Switzerland

**Heike Scheithauer**

Germany

**Hans Schieferr**

Switzerland

**Alexander Schlaefer**

Germany

**Michel Schlienger**

France

**Maximilian Schmid**

Austria

**Manfred Schmidt**

Germany

**Uwe Schneider**

Switzerland

**Max Schnurr**

Germany

**Michael Scholz**

Germany

**David Schreiber**

United States of America

**Ulrich Schüller**

Germany

**Amanda Schwint**

Argentina

**Marta Scorsetti**

Italy

**Clemens Seidel**

Germany

**Robert Semrau**

Germany

**Mehmet Sen**

United Kingdom

**Matteo Seregni**

Italy

**Marco Serpa**

Germany

**Waseem Sharieff**

Canada

**Gregory Sharp**

United States of America

**Yuta Shibamoto**

Japan

**Keiko Shibuya**

Japan

**Helen Shih**

United States of America

**Kyung Hwan Shin**

South Korea

**Yoshiyuki Shioyama**

Japan

**Ori Shokek**

United States of America

**David Shultz**

United States of America

**Frank-André Siebert**

Germany

**Nanna Sijtsema**

Netherlands

**Charles Simone**

United States of America

**Anurag Singh**

United States of America

**Ben Slotman**

Netherlands

**Wendy Smith**

Canada

**Gregory Smyth**

United Kingdom

**Toshinori Soejima**

Japan

**Riccardo Soffietti**

Italy

**Shiyu Song**

United States of America

**Akinaga Sonoda**

Japan

**Yukihiko Sonoda**

Japan

**Ute Spahn**

Germany

**Hanno M. Specht**

Germany

**Lena Specht**

Denmark

**Michael Spiotto**

United States of America

**Daniel Spratt**

United States of America

**Michael Stahl**

Germany

**Lukas Stalpers**

Netherlands

**Roel Steenbakkers**

Netherlands

**Diana Steinmann**

Germany

**Kevin Stephans**

United States of America

**Florian Stieler**

Germany

**Alison Stillie**

United Kingdom

**Sandro Stoeckli**

Switzerland

**Igor Stojkovski**

Macedonia

**Markus Stoll**

Germany

**Bjoern Stork**

Germany

**Guy Storme**

Belgium

**Tino Streller**

Switzerland

**Lidia Strigari**

Italy

**Vratislav Strnad**

Germany

**Carmen Stromberger**

Germany

**Gabriela Studer**

Switzerland

**Martin Stuschke**

Germany

**Shinji Sugahara**

Japan

**Xiaonan Sun**

China

**Katarina Surlan Popovic**

Slovenia

**Clare Suttie**

Australia

**Phillip Taddei**

Lebanon

**Takeo Takahashi**

Japan

**Wataru Takahashi**

Japan

**Tetsuo Takehara**

Japan

**Ozgur Tanriverdi**

Turkey

**Mohmad Ashraf Teli**

India

**Chris Terhaard**

Netherlands

**Wolfgang Thasler**

Germany

**David Thomas**

United States of America

**CR Thomas**

United States of America

**Daniela Thorwarth**

Germany

**Zhen Tian**

United States of America

**Carmen Timke**

Germany

**Ingeborg Tinhofer**

Germany

**Marcello Tiseo**

Italy

**Florent Tixier**

France

**Manuel Todorovic**

Germany

**Yuji Toiyama**

Japan

**Koichi Tokuuye**

Japan

**Vincenzo Tombolini**

Italy

**Erkan Topkan**

Turkey

**Dunyaporn Trachootham**

Thailand

**Alison Tree**

United Kingdom

**Silke Tribius**

Germany

**Theresa Trotter**

Canada

**Pauline Truong**

Canada

**Erik Tryggestad**

United States of America

**James Tucker**

United States of America

**Sandra Turner**

Australia

**Matthias Uhl**

Germany

**Kristian Unger**

Germany

**Dmytro Unukovych**

Sweden

**Maurizio Valeriani**

Italy

**Nicholas Van As**

United Kingdom

**Tara Van de Water**

Netherlands

**Wouter Van Elmpt**

Netherlands

**Dirk Van Gestel**

Belgium

**Paul Van Houtte**

Belgium

**Hilde Van Parijs**

Belgium

**Joep Van Roermund**

Netherlands

**John Randolf van Sornsen de Koste**

Netherlands

**Jef Vandemeulebroucke**

Belgium

**Hansjoerg Vees**

Switzerland

**Vaneja Velenik**

Slovenia

**Karen Venables**

United Kingdom

**Wilko Verbakel**

Netherlands

**Dirk Verellen**

Belgium

**Frank Verhaegen**

Netherlands

**Marcel Verheij**

Netherlands

**Daniel Vetterli**

Switzerland

**Gregory Videtic**

United States of America

**Asgaut Viste**

Norway

**Dirk Vordermark**

Germany

**Wan Zamaniah Wan Ishak**

Malaysia

**Hesheng Wang**

United States of America

**Ping Wang**

China

**Po-Ming Wang**

Taiwan

**Luhua Wang**

China

**Fredrik Wärnberg**

Sweden

**Ronald Warnick**

United States of America

**Samantha Warren**

United Kingdom

**Yoichi Watanabe**

United States of America

**Damien Charles Weber**

Switzerland

**Christian Weiss**

Germany

**Stefan Welz**

Germany

**Thomas G. Wendt**

Germany

**Rene Werner**

Germany

**John Werning**

United States of America

**Charlotte Westbury**

United Kingdom

**Greg Wheeler**

Australia

**Joachim Widder**

Netherlands

**Tilo Wiezorek**

Germany

**Theo Wiggers**

Netherlands

**Bas Wijnhoven**

Netherlands

**Christopher Willett**

United States of America

**Christopher Willey**

United States of America

**Lucy Wills**

United Kingdom

**Joerg Wischhusen**

Germany

**Barbara Witulla**

Germany

**Ulrich Wolf**

Germany

**Jens Wölfelschneider**

Germany

**Hendrik Andreas Wolff**

Germany

**Binbin Wu**

United States of America

**Q. Jackie Wu**

United States of America

**Jun-xin Wu**

China

**Qiuwen Wu**

United States of America

**Abraham Wu**

United States of America

**Florian Wuerschmidt**

Germany

**Evan Wuthrick**

United States of America

**Zhong-jun Xia**

China

**Lei Xing**

United States of America

**Ting Xu**

United States of America

**Yoshiya Yamada**

United States of America

**Jack Yang**

United States of America

**Kamil Yenice**

United States of America

**Kheng-Wei Yeoh**

Singapore

**Ugur YILMAZ**

France

**Yong Yin**

China

**Hongmei Ying**

China

**Indra Yohannes**

Germany

**Young Do Yoo**

South Korea

**John Yordy**

United States of America

**Ken Yoshida**

Japan

**Cedric Yu**

United States of America

**James Yu**

United States of America

**Ning Yue**

United States of America

**Nikolaos Zamboglou**

Germany

**Tomas Zaremba**

Denmark

**Paul Zarogoulidis**

Greece

**Xin Zhang**

United States of America

**Zhen Zhang**

China

**Weiling Zhao**

United States of America

**Chong Zhao**

China

**Dandan Zheng**

United States of America

**Jinyuan Zhou**

United States of America

**Li Zhou**

China

**Hong Zhou**

China

**Thomas Zilli**

Switzerland

**Zvonimir Zore**

Croatia

**Zachary Zumsteg**

United States of America

**Brigitte Zurl**

Austria

**Daniel Zwahlen**

Switzerland

**Felix Zwicker**

Germany

